# Complete Genomes of Symbiotic Cyanobacteria Clarify the Evolution of Vanadium-Nitrogenase

**DOI:** 10.1093/gbe/evz137

**Published:** 2019-06-27

**Authors:** Jessica M Nelson, Duncan A Hauser, José A Gudiño, Yessenia A Guadalupe, John C Meeks, Noris Salazar Allen, Juan Carlos Villarreal, Fay-Wei Li

**Affiliations:** 1 Boyce Thompson Institute, Ithaca, New York; 2 Plant Biology Section, Cornell University, Ithaca, New York; 3 Smithsonian Tropical Research Institute, Panama City, Panama; 4 Department of Microbiology and Molecular Genetics, University of California, Davis, California; 5 Department of Biology, Laval University, Quebec City, Quebec, Canada

**Keywords:** *Nostoc*, *Blasia*, hornworts, liverworts, phylogenomics, nanopore

## Abstract

Plant endosymbiosis with nitrogen-fixing cyanobacteria has independently evolved in diverse plant lineages, offering a unique window to study the evolution and genetics of plant–microbe interaction. However, very few complete genomes exist for plant cyanobionts, and therefore little is known about their genomic and functional diversity. Here, we present four complete genomes of cyanobacteria isolated from bryophytes. Nanopore long-read sequencing allowed us to obtain circular contigs for all the main chromosomes and most of the plasmids. We found that despite having a low 16S rRNA sequence divergence, the four isolates exhibit considerable genome reorganizations and variation in gene content. Furthermore, three of the four isolates possess genes encoding vanadium (V)-nitrogenase (*vnf*), which is uncommon among diazotrophs and has not been previously reported in plant cyanobionts. In two cases, the *vnf* genes were found on plasmids, implying possible plasmid-mediated horizontal gene transfers. Comparative genomic analysis of *vnf*-containing cyanobacteria further identified a conserved gene cluster. Many genes in this cluster have not been functionally characterized and would be promising candidates for future studies to elucidate V-nitrogenase function and regulation.

## Introduction

Symbiotic interactions with microbes play a pivotal role in eukaryote evolution. In plants, microbial symbionts produce growth-promoting substances, boost tolerance to environmental stress, and can greatly expand access to essential nutrients (Lugtenberg and Kamilova 2009). Partnerships with nitrogen-fixing bacteria are of particular importance because they relieve plants from nitrogen limitation. The best-known example is the symbiosis between legumes and rhizobia, which is an area of active investigation aiming to transfer the symbiosis to nonlegume crop plants (Mus et al. 2016).

There is another and often overlooked type of N_2_-fixing symbiosis in which certain plants partner with heterocyst-forming cyanobacteria (supplementary fig. 1, Supplementary Material online). Compared with rhizobia, they are less dependent on the plant partner—they have their own specialized cells (i.e., heterocysts) for N_2_-fixation and do not require hosts to create a low-oxygen environment—and therefore hold a promising translational potential for installing N_2_-fixation into crop plants. In addition, unlike the legume-rhizobia symbiosis, the plant-cyanobacteria endosymbiotic partnership has evolved independently multiple times—in two lineages of bryophytes (liverworts and hornworts; supplementary fig. 1, Supplementary Material online), ferns (*Azolla*), gymnosperms (cycads), and a genus of angiosperms (*Gunnera*) (Adams et al. 2006), thereby offering a unique opportunity to study convergent evolution of plant–microbe symbiosis.

The key function of cyanobacteria in plant symbiosis is nitrogen fixation, which is carried out by nitrogenase. Nitrogenase is a two-component protein complex consisting of dinitrogenase and dinitrogenase reductase. The most common nitrogenase uses molybdenum (Mo) as the cofactor; however, certain cyanobacteria and other diazotrophs possess an “alternative” nitrogenase that uses vanadium (V) instead of Mo (Mus et al. 2018). Although structurally similar, Mo- and V-nitrogenases are encoded by separate gene clusters and have different catalytic properties; for example, Mo-nitrogenase is more efficient above 16 °C whereas V-nitrogenase performs better at a lower temperature (Miller and Eady 1988). Additionally, Mo is more scarce than V in the terrestrial environment (Wedepohl 1995) and can be a limiting factor for biological nitrogen fixation (Rousk et al. 2017). It is therefore likely that having both nitrogenases could be advantageous for diazotrophs to fine-tune nitrogen fixation in response to mineral availability and temperature. However, although the existence of V-nitrogenase in cyanobacteria is known, its phylogenetic distribution is unclear, and its role in plant symbiosis has not been explored.

As part of our ongoing effort to probe the diversity of plant symbiotic cyanobacteria, we assembled four complete circular genomes of cyanobacteria isolated from hornworts and a liverwort. We report here the discovery of V-nitrogenase in plant cyanobionts, and present comparative genomic analyses to shed new light on the distribution and evolution of V-nitrogenase.

## Materials and Methods

### Isolation and Culture of Cyanobionts

Symbiotic cyanobacteria were cultured from the hornworts *Phaeoceros carolinanus* and *Leiosporoceros dussii*, as well as the liverwort *Blasia pusilla* (table 1). For *Blasiapusilla* and *P. carolinianus*, gametophyte thalli were dissected to obtain pieces with one cyanobacterial colony each. These fragments were wetted in 1% Tween 20, surface sterilized with 5% bleach solution (that has 6% sodium hypochlorite) for 2 min, and rinsed three times in sterile water. For *Leiosporocerosdussii*, cleaned thalli were sterilized in a 10% bleach solution for 1 min and washed three times with sterile water (1 min each). One-millimeter (in thickness) sections from the center of thalli were cut and placed in microporous capsules that were partially submerged in sterile water. Transverse sections of thalli were sterilized for the second time, by submerging the microporous capsules in a 10% bleach solution for 30 s and washing them in three changes of sterile water, 1 min each.

**Table 1:** Cyanobacteria Strains, Nanopore Sequencing, and Assembly Statistics

Strain	Taxonomic Assignment	Host Species	Locality	Raw Reads (Mb)	Raw Read Length N50 (kb)	Filtered Reads (Mb)	Filtered Read Length N50 (kb)	Assembled Chromosome Size (Mb)	Assembled Plasmids (Mb)
C52	*Nostoc* sp.	*Blasia pusilla*	USA, New York, Adirondacks	8,050	4,380	847	12,147	7.83c	0.54^c^, 0.34^c^, 0.30^c^, 0.28^c^, 0.23^c^, 0.22^c^, 0.10^l^, 0.04^l^
C57	*Nostoc* sp.	*Phaeoceros carolinianus*	USA, California, Mendocino Co.	253	3,662	204	5,098	7.50^c^	0.24^c^, 0.16^c^, 0.06^c^, 0.06^c^, 0.06^c^, 0.04^c^, 0.33^l^, 0.32^l^, 0.16^l^, 0.13^l^, 0.08^l^, 0.03^l^, 0.03^l^, 0.02^l^, 0.02^l^
TCL240-02	*Nostoc* sp.	*Leiosporoceros dussii*	Panamá, Río Guayabo, El Valle de Antón.	960	7,764	587	14,348	7.89^c^	NA
TCL026-01	*Nostoc* sp.	*Leiosporoceros dussii*	Panamá, Río Guayabo, El Valle de Antón.	3,640	2,420	421	15,235	6.77^c^	0.22^c^, 0.14^c^, 0.11^c^, 0.05^c^, 0.04^c^, 0.04^c^, 0.03^c^

Note.— ^c^Circular assembly and ^l^linear assembly.

Surface-sterilized thallus fragments were put on combined nitrogen-free BG11 medium (BG11_0_; Rippka et al. 1979), solidified with 1% (w/v) agar and incubated at 22 °C under a 12-h day/night cycle. After growth was detected on the BG11_0_ plates, cultures were transferred to liquid BG11_0_ and grown with continuous shaking under the same incubation conditions. Cultures were checked for contamination by fungi and heterotrophic bacteria by streaking on 1.5% malt extract agar and lysogeny broth agar, respectively, and incubating at room temperature in the dark. No sign of contamination was found for these four cultures after at least 10 days of incubation.

### DNA Extraction

Aliquots of liquid cultures of cyanobacteria were centrifuged at 17,000 × g for 2 min to pellet the cells, liquid medium was removed, and one copper bead was added to each pellet. The tubes holding the cell pellets were frozen in liquid nitrogen and ground for 1 min at 1,300 strokes/min on a SPEX SamplePrep 1600 MiniG tissue homogenizer. The metal rack holding the tubes was also submerged in liquid nitrogen before grinding to ensure the samples stayed cold during disruption. Pellets were resuspended in prewarmed 2× cetyl trimethylammonium bromide (CTAB) with 1% β-mercaptoethanol and incubated at 65 °C for 1 h, with gentle mixing every 15 min. Samples were extracted with an equal volume of 24:1 chloroform:isoamyl alcohol twice, each consisting of 5 min gentle mixing on a Labnet Mini Labroller and 5 min centrifugation at 17,000 × g. Wide bore pipette tips were used to transfer the aqueous layer in order to maintain DNA integrity. DNA was precipitated with an equal volume of isopropanol and pelleted for 30 min at 17,000 × g and 4 °C. The DNA pellets were washed twice with 70% ethanol, air dried in a sterile air hood, resuspended in Tris–HCl pH 7.5, and treated with RNase for 1 h at 37 °C.

### Nanopore and Illumina Sequencing

Nanopore libraries were prepared by using the Ligation Sequencing Kit (SQK-LSK109) in conjunction with the Native Barcoding Expansion Kit (1-12). No size selection was done prior to the library preps. We sequenced the four libraries on two MinION R9 flowcells (strains C52 + TCL26-01 on one and C57 + TCL240-02 on the other). Sequencing duration was set to 60 h. We basecalled the raw data using the “flip-flop” algorithm in guppy (version 2.3.7).

For Illumina library construction, we used the SparQ DNA Frag & Library Prep kit and Adapter Barcode Set A following the manufacturer’s protocol. Approximately 100 ng DNA from each strain was used as the input, and fragmented for 5 min in the Fragmentation mix. Libraries were amplified with eight polymerase chain reaction cycles, followed by size selection at 300–700 bp using sparQ PureMag Beads. The four cyanobacteria libraries were pooled with eight other samples in equal quantity and sequenced together on an Illumina NexSeq500 mid-output flowcell at Cornell Institute of Biotechnology.

### Genome Assembly and Annotation

Basecalled nanopore reads were first filtered by length: a 10-kb cutoff was applied to strains C52 and TCL026-01, 5 kb to TCL240-02, and 1 kb to C57. We used Flye (Kolmogorov et al. 2019) for genome assembly with five iterations of built-in polishing, followed by four rounds of minimap2-Racon (Li 2016; Vaser et al. 2017) and one round of medaka (https://github.com/nanoporetech/medaka; last accessed April 2019) error correction steps. For strain C57, because we were unable to obtain a circular chromosome after Flye assembler, circlator (Hunt et al. 2015; with the option “–assembler spades –split_all_reads –merge_min_id 85 –merge_breaklen 1000”) was used to merge and circularize contigs. The final nanopore assemblies were further polished by Illumina reads with four iterations of bwa-Pilon (Li and Durbin 2009; Walker et al. 2014). Genome annotations were done using prokka (Seemann 2014), with a custom *Nostoc* database compiled from GenBank.

### Phylogenetic Analyses

We first reconstructed a preliminary 16S phylogeny including our sequenced strains and all the cyanobacteria genomes available from GenBank. From this phylogeny, we then selected 96 genomes that are related to our cyanobiont isolates or have *vnf* genes (supplementary table 1, Supplementary Material online). To build a phylogenomic data set, we used BUSCO (Benchmarking Universal Single-Copy Orthologs; Simão et al. 2015) to extract conserved, single-copy genes. A total of 834 cyanobacterial BUSCO loci (“cyanobacteria_odb9”) were searched against the genomes, and sequences annotated as “fragment” or “duplicated” were excluded from the data set. Each BUSCO locus was aligned separately by MAFFT (Katoh et al. 2002). The final concatenated alignment contains 326,259 amino acid sites (available as supplementary materials, Supplementary Material online). To infer a phylogenetic relationship, a maximum likelihood analysis was carried out by RAxML with the JTT substitution model (Stamatakis 2014), and a total of 1,000 rapid bootstraps were done to assess the branch support.

### Comparative Genomic Analyses

To infer large-scale genomic rearrangements among the four sequenced strains, we used the jcvi.compara.synteny function in the package MCscan (Tang et al. 2008; with the option “screen –minspan = 15”) to identify and visualize syntenic blocks. For the finer-scale syntenic relationship around the *vnf* genes, the same MCscan function was used but with “mcscan –iter = 1.” To infer the degree of gene content conservation among the four genomes, Orthofinder2 (Emms and Kelly 2018) was used to classify gene orthogroups. The numbers of shared orthogroups were then plotted by UpSetR (Conway et al. 2017).

## Results and Discussion

### Cyanobiont Genome Assemblies

**Fig. 1.**
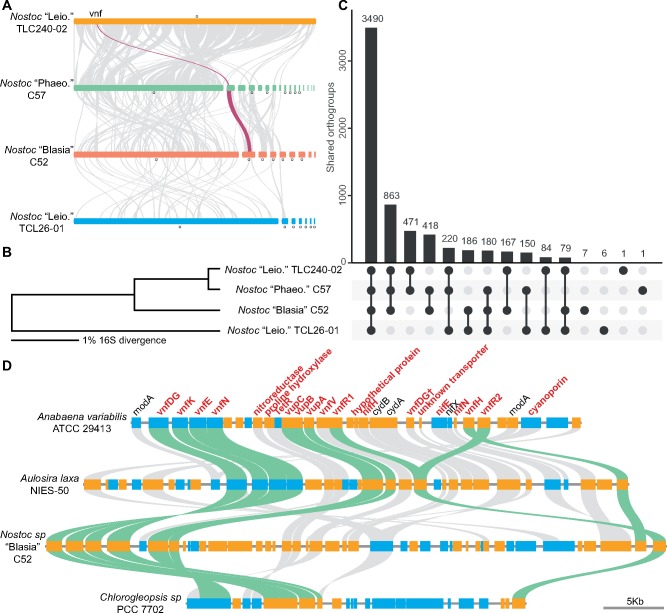
—Comparative analyses of cyanobacteria genomes. (*A*) Considerable differences in genome organization were found among the four cyanobiont genomes sequenced in this study. Ribbons connect the syntenic blocks, with red ribbons marking the location of the *vnf* gene cluster. Note the contig lengths are not to scale. (*B*) The four cyanobiont strains show very little 16S sequence divergence. (*C*) Although the majority of the orthogroups are shared among the four genomes, gains and losses appear to be common. “Leio.”: strain isolated from *Leiosporoceros dussii*; “Phaeo.”: from *Phaeoceros carolinianus*; “Blasia”: from *Blasia pusilla*. “o” indicates circular chromosomes or plasmids. (*D*) A conserved *vnf*-associated gene cluster was found in cyanobacteria. Genes shared among at least three genomes are labeled in red. Green ribbons connect genes that have been functionally characterized in *Anabaena variabilis* ATCC 29413. Blue and orange gene blocks distinguish the transcription directions. “†” indicates a truncated gene in *Anabaena variabilis*.

Although genomes have been published for several plant cyanobionts (Meeks et al. 2001; Ran et al. 2010; Li et al. 2018; Kanesaki et al. 2018; Warshan et al. 2018; Gutiérrez-García et al. 2019), only *Nostoc punctiforme* PCC 73102 (from a cycad; Meeks et al. 2001) and *Nostoc azollae* (from *Azolla*; Ran et al. 2010) have genomes assembled into complete circular chromosomes. All others are highly fragmented with hundreds to thousands of contigs, making comparative genomic studies difficult. In this study, even with nanopore coverage as low as ∼20× (table 1), we were able to obtain circular contigs for all four main chromosomes and for most of the plasmids (fig. 1*a*; table 1). However, we found that our nanopore assemblies all have a low nucleotide accuracy. After multiple rounds of Racon and medaka correction, over half of the BUSCO genes were still missing or fragmented (supplementary fig. 2, Supplementary Material online), often due to frameshifts introduced by inaccurate homopolymer resolution. Further polishing using Illumina reads was required to bring BUSCO genes to >99% completion (supplementary fig. 2, Supplementary Material online). This indicates that the error profile of nanopore reads is not random, and further development is needed so that one could bypass the Illumina data generation step.

### Variation in Genome Organization and Gene Content

Comparing the four newly sequenced genomes, we found that the level of 16S sequence divergence is low, ranging from 6.33% to 0.34% (fig. 1*b*). This is a stark contrast to the large-scale genomic rearrangements identified by synteny analysis (fig. 1*a*), as well as the relatively low number of shared orthogroups (fig. 1*c*). For example, even though 16S sequences of TCL240-02 and C57 are 99.66% identical, the two genomes have very different genome organizations and plasmid numbers (fig. 1*a*). Furthermore, over 17% of the orthogroups are present in only one of two genomes (fig. 1*c*). Although 16S is a commonly used marker to profile microbial communities, the results shown here imply that 16S could seriously underestimate the diversity at the genomic (and presumably functional) level, especially when a less stringent cutoff (e.g., 97%) is applied for clustering Operational Taxonomic Units.

### Phylogenetic Relationship of Symbiotic Cyanobacteria

Overall, our phylogeny shows that a large number of cyanobacteria genera are polyphyletic with respect to morphology-based taxonomic identity (supplementary fig. 3, Supplementary Material online), which is consistent with several recent studies (Herdman and Rippka 2017; Gagunashvili and Andrésson 2018; Warshan et al. 2018; Will et al. 2019). When placed under a broader phylogenetic context, our sequenced strains fell into two clades (supplementary fig. 3, Supplementary Material online). Clade A is mostly comprised of cyanobacterial endosymbionts of lichens, bryophytes, and a cycad, as well as those found as epiphytes on boreal feather mosses (Warshan et al. 2018). This clade corresponds to the “Intracellular/Extracellular” group in a recent phylogenomic study by Warshan et al (2018). Clade B on the other hand, has fewer strains isolated from plants or lichens, although symbiotic competency has not been tested for most of the strains in this clade. Warshan (2018)’s “Extracellular II” group is nested within this clade.

### Presence of Vanadium-Type Nitrogenase in Plant Cyanobionts

Three of the four cyanobacteria genomes we assembled contain the genes encoding vanadium (V)-nitrogenase (*vnf*), in addition to the conventional Mo-type nitrogenase. Although *vnf* genes have been previously identified in cyanobacteria associated with lichens (Hodkinson et al. 2014; Darnajoux et al. 2017; Gagunashvili and Andrésson 2018), this is the first report, to our knowledge, of them in plant cyanobionts. Interestingly, in C57 and C52, *vnf* genes are located in plasmids (fig. 1*a*), implying there is a potential for plasmid-mediated horizontal gene transfers. To better understand the distribution of *vnf* in cyanobacteria, we mined the NCBI database and identified four separate cyanobacteria lineages having *vnf* (Clades A–D; see the red taxa in supplementary fig. 3, Supplementary Material online). None of these clades are exclusively composed of *vnf*-containing strains, and it appears that *vnf* losses and/or gains are frequent among cyanobacteria. For strains having complete circular genomes, only strains C57 and C52 have *vnf* in plasmids.

### A Conserved *vnf* Cluster in Cyanobacteria

V-nitrogenase was originally discovered in *Azotobacter* (Robson et al. 1986) and subsequently identified and characterized in the cyanobacterium *Anabaena variabilis* ATCC 29413 (Thiel 1993; Thiel et al. 2014). In *Anabaenavariabilis* ATCC 29413, *vnf* genes form a cluster consisting of *vnfDGK* (nitrogenase structural proteins; Thiel 1993), *vnfH* (dinitrogenase reductase; Pratte et al. 2006), and *vnfEN* (for cofactor synthesis; Thiel 1996). Additionally, genes encoding vanadate transporters (*vupABC*; Pratte and Thiel 2006) and *vnf* transcriptional regulators (*vnfR1* and *R2*; Pratte et al. 2013) have also been identified in a close proximity to *vnf* genes. To determine if this gene cluster is conserved across the four *vnf*-containing clades (Clades A–D; supplementary fig. 3, Supplementary Material online), we carried out a detailed microsynteny analysis with one representative genome from each clade. We were able to delineate a clear conserved gene cluster associated with V-nitrogenase (fig. 1*d*). This cluster includes not only genes with known functions such as *vnfR2* and *vupABC* but also a large number of uncharacterized genes (fig. 1*d*). Whether these conserved genes are involved in V-nitrogenase activity is unresolved, but our finding here provides a list of promising candidates to analyze.

## Supplementary Material

Supplementary data are available at *Genome Biology and Evolution* online.

## Supplementary Material

Supplementary_Matrial_evz137

## Notes

**Data deposition:** This project has been deposited at NCBI BioProject under the accession PRJNA534312.
