# Supplementary material for: Complete Genomes of Symbiotic Cyanobacteria Clarify the Evolution of Vanadium-Nitrogenase
Source: Genome Biol Evol. 2019 Jun 27;11(7):1959–64. doi: 10.1093/gbe/evz137 (PMC6645180; doi:10.1093/gbe/evz137)
Supplement: Supplementary_Matrial_evz137 [file supplementary_matrial_evz137.zip › Supplementary_Figures.pdf]

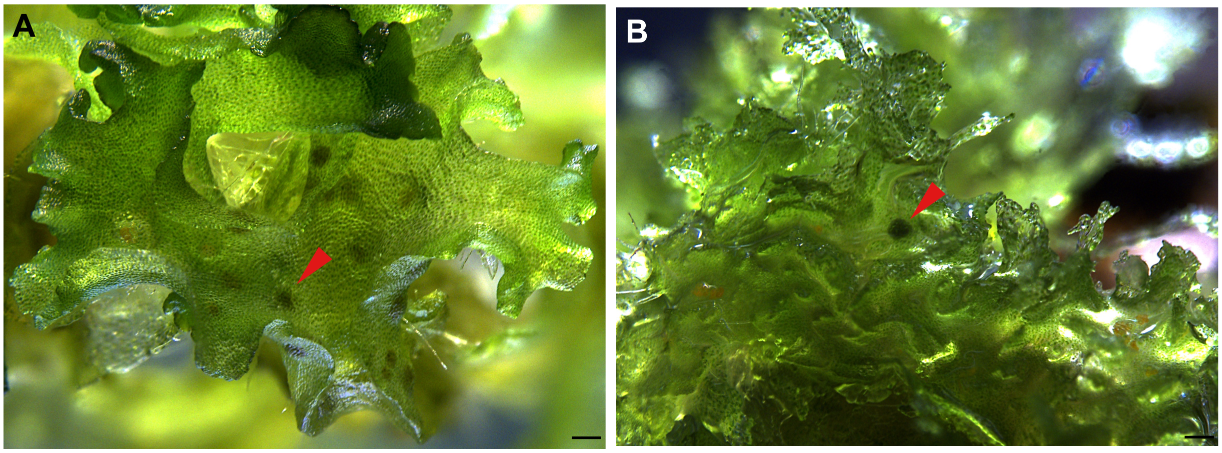

Supplementary Figure 1. **Symbiotic interaction between cyanobacteria and hornworts.** *in vitro* reconstituted symbiosis between (A) cyanobacteria strain C57 and *Phaeoceros carolinianus,* and (B) strain C52 and *Anthoceros agrestis*. Red arrowhead points to a cyanobacteria colony.

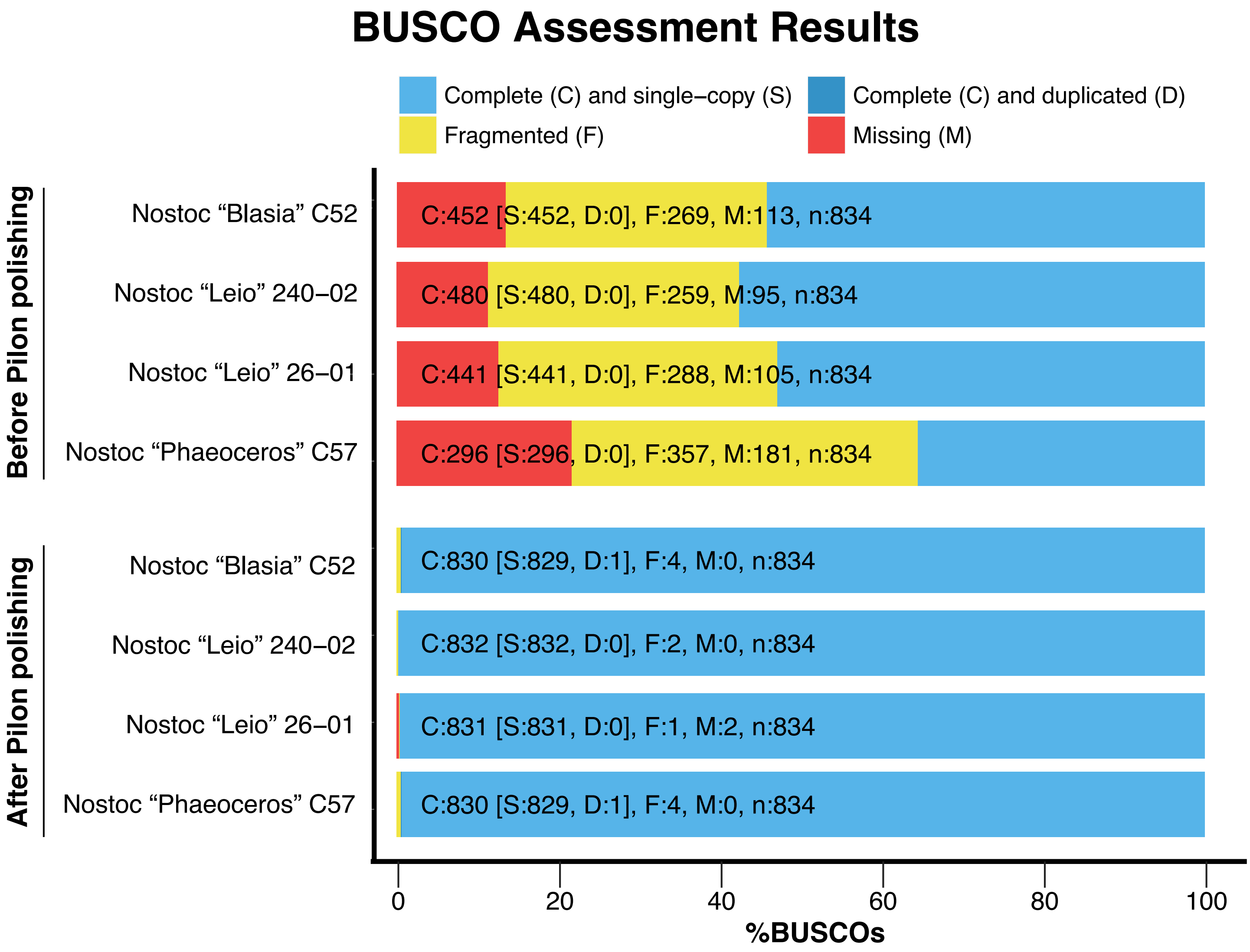

Supplementary Figure 2. **Nanopore assemblies have low nucleotide accuracy and require Illumina polishing.**

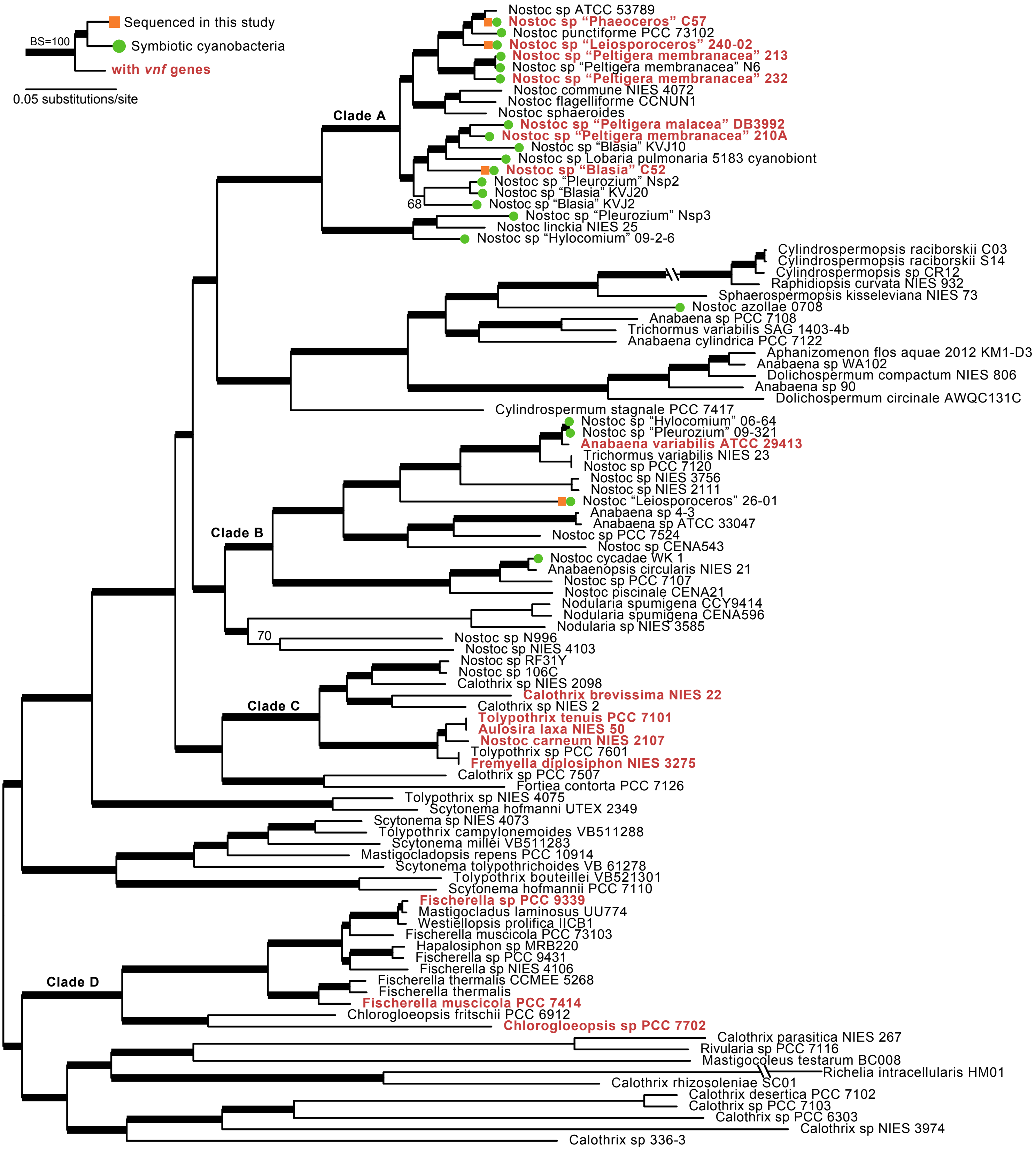

Supplementary Figure 3. **Phylogenetic relationship of 100 cyanobacteria genomes.** The maximum likelihood tree was based on 834 BUSCO single-copy genes. Thickened branches indicate bootstrap value of 100. Genomes colored in red contain vnf genes, green circles mark symbiotic strains isolated from plants or lichens, orange squares indicate the genomes sequenced in this study.
